# Evolutionary Analysis and Antiviral Drug Prediction of Mpox Virus

**DOI:** 10.3390/microorganisms12112239

**Published:** 2024-11-05

**Authors:** Zhong-Hao Lian, Chen-Hui Yang, Ye Qiu, Xing-Yi Ge

**Affiliations:** Hunan Provincial Key Laboratory of Medical Virology, College of Biology, Hunan University, Changsha 410012, China; lianzhonghao@hnu.edu.cn (Z.-H.L.); yangch@hnu.edu.cn (C.-H.Y.); qiuye@hnu.edu.cn (Y.Q.)

**Keywords:** MPXV, positive selection, MIHP, alvespimycin, imiquimod

## Abstract

The resurgence of mpox virus (MPXV) poses a significant challenge to global public health. Currently, there is a limited understanding of the evolutionary details of MPXV during its epidemics, and no specific drugs have been developed for it. Herein, analysis of mutations and positive selection sites (PSSs) within the MPXV genomes revealed 799 mutations and 40 PSSs. Visualization analysis indicated that these mutations and PSSs may affect protein structure. Additionally, a protein–protein interaction (PPI) network between human and MPXV was established, identifying 346 MPXV-interacting human proteins (MIHPs). An interaction network involving MIHPs and other viruses confirmed that these proteins can interact with various viruses that infect humans. Functional analysis of MIHPs suggested their enrichment in host immunity pathways. Lastly, two drugs targeting MIHPs and four compounds targeting MPXV proteins were screened as candidate antivirals against MPXV. These findings not only deepen our understanding of MPXV evolution but also aid in the development of anti-MPXV drugs.

## 1. Introduction

The human mpox disease (MPX) is a zoonotic disease caused by the mpox virus (MPXV). Typical symptoms of MPX include fever, headache, lymphadenopathy, and cutaneous rashes and blisters [1]. MPXV belongs to the Orthopoxvirus genus, which also includes variola virus (VARV), vaccinia virus (VACV), and cowpox virus (CPXV) [2]. In 1958, MPXV was first isolated from the pustules of monkeys at the Statens Serum Institute in Denmark [3]. An imported case of MPXV was reported in the United Kingdom in May 2022, followed by outbreaks worldwide [4]. From May 2022 to May 2023, there were >87,500 cases and 141 deaths reported (https://worldhealthorg.shinyapps.io/mpx_global, accessed on 31 October 2024). The 2022 outbreak attracted global attention due to MPXV transmission in non-endemic areas and through sexual contact. The MPXV cases of 2022 outbreak were predominantly detected in homosexuals, especially in Europe and other non-African countries.

MPXV has two distinct genetic branches: Clade I and Clade II. Clade I is predominantly epidemic in Central Africa and is associated with severe clinical symptoms and a higher mortality rate. Clade II is mainly confined to West Africa, resulting in milder symptoms and lower mortality [5]. Clade II includes sub-Clades IIa and IIb, with Clade IIb evolving from Clade IIa. Clade IIb is further subdivided into several sub-lineages based on their phylogenetic relationship, such as Clade IIb A.1, Clade IIb A.2, and Clade IIb B [6]. Historically, Clade I has predominated, while Clade IIb B.1 was the predominant strain during the 2022 outbreak [7,8].

Different strains cause different symptoms. In the non-human primate models, Clade II replicated only in the skin and lymphatic system, while Clade I may affect the respiratory, gastrointestinal, and urogenital tracts [9]. The symptoms of Clade I and Clade II include a pronounced prodromal phase, generalized rash, cervical or axillary lymphadenopathy, frequent proctitis, and urethritis and pharyngitis, while the symptoms of Clade IIb include an unpronounced prodromal phase, localized rash (especially in the anus), and inguinal lymphadenopathy [10]. In some recent cases of Clade Ib, the patient had a more swollen throat that was accompanied by severe pain (https://www.bbc.com/news/articles/cly3xzdq909o, accessed on 31 October 2024).

MPXV transmission slowed after it was reported in August 2022 [11], and by April 2024, Clade IIb B.1 had almost subsided (https://worldhealthorg.shinyapps.io/mpx_global/, accessed on 15 September 2024). However, as the Clade II outbreak waned, cases of Clade I infections in Central Africa, particularly in the Republic of Congo and the Central African Republic, began to increase [12]. On 15 August 2024, the Clade I variant was detected in Sweden, and on 22 August, Thailand reported the presence of Clade Ib. Clade Ib appears to differ in that it can spread through human-to-human contact, including sexual contact [13]. Among the provinces in the Republic of the Congo with reported MPXV cases in 2024, 50% of those cases were individuals under 15 years old, indicating increased vulnerability among children to Clade I (https://www.who.int/emergencies/disease-outbreak-news/item/2024-DON522, accessed on 20 September 2024). Consequently, the WHO Director-General declared the MPX outbreak a public health emergency of international concern (PHEIC) on 14 August 2024 (https://worldhealthorg.shinyapps.io/mpx_global/, accessed on 15 September 2024). As of 31 July 2024, over half a century of transmission, there have been 102,997 confirmed cases of MPXV and 223 reported deaths (https://worldhealthorg.shinyapps.io/mpx_global/, accessed on 15 September 2024). MPXV caused significant damage to global public health [14].

MPXV is a type of double-stranded DNA (dsDNA) virus [15]. Its genome size is approximately 200 kbp, encoding about 190 proteins, which contributes to MPXV’s wide host range and complex immune escape mechanisms [16,17]. The regions from NBT03_gp036 (F12L) to NBT03_gp130 (A24R) in the MPXV genome represent the core regions, while the remainder consists of the variable regions [18]. Despite the inherent stability of a dsDNA genome, MPXV exhibited a nucleotide substitution rate during the 2022 outbreak that was six to twelve times higher than in previous instances, along with a significant number of mutations [19,20]. Previous research indicated that Clade IIb B.1 has been undergoing micro-evolution due to point mutations during transmission [21]. Additionally, a new variant of Clade I, primarily carrying APOBEC3-type mutations, was reported in South Kivu, suggesting that MPXV is adapting as it spreads among humans (https://www.who.int/emergencies/disease-outbreak-news/item/2024-DON522, accessed on 20 September 2024). Given the rapid evolution of MPXV, a comprehensive analysis of mutations and positive selection is essential for understanding its evolution.

Based on nucleic acid amplification testing (NAAT), MPXV infection is currently confirmed primarily by real-time or conventional polymerase chain reaction (PCR) (https://www.who.int/publications/i/item/WHO-MPX-Laboratory-2024.1, accessed on 31 October 2024). Antiviral drugs play a significant role in the prevention and control of viral diseases [22]. However, there are currently no effective commercial antiviral drugs or vaccines specifically for MPXV [23]. Due to the genetic similarities between MPXV and VARV, candidate drugs for MPXV have been selected from those used against VARV, including cidofovir, brincidofovir, and tecovirimat [24]. However, these drugs may cause a variety of adverse reactions, for instance, tecovirimat may lead to dizziness, retching, and skin swelling [25]. Thus, the safety and effectiveness remain to be validated, urging the need to develop more candidates for MPXV-specific medications. Compared to de novo drug development, repurposing existing drugs is an effective strategy for treating diseases [26]. Researchers have utilized protein–protein interaction (PPI) networks to predicted antiviral drugs from existing medications. For example, a study reported a PPI network to predict candidate drugs against EV71 and verified their antiviral effect [27]. Currently, a large number of PPIs between viruses and their hosts have been reported. Predicting drugs through PPI networks can significantly contribute to the development of treatments against MPXV.

In this study, we analyzed the MPXV genome, identifying 799 mutations and 40 positive selection sites (PSSs). Our visualization analysis suggested that these mutations and PSSs may influence protein structure. Additionally, we constructed a network of MPXV–human protein–protein interactions (PPIs), identifying 346 MPXV-interacting human proteins (MIHPs). We also developed networks for drug–MIHP interactions and drug–MPXV protein interactions to predict potential drugs against MPXV. By evaluating druglikeness, molecular docking, and immunogenicity, we identified two drugs and four compounds. These two drugs targeting MIHPs may have a relatively broad-spectrum antiviral potential, and they are more stable and less susceptible to drug resistance.

## 2. Materials and Methods

### 2.1. Research Design and Work Flow

**Figure 1:**
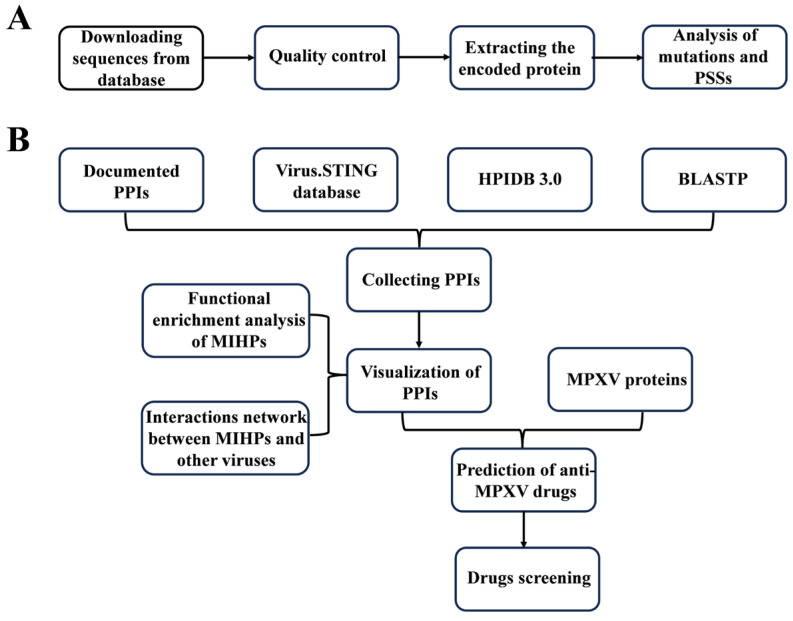
A schematic of the workflow of this study. (**A**) The analysis of mutations and PSSs within the MPXV genome. (**B**) Anti-MPXV drug screening. MPXV, mpox virus; PSSs, positive selection sites; PPIs, protein–protein interactions; MIHPs, MPXV-interacting human proteins.

The design of this study includes two parts: analysis of mutations and PSSs within the MPXV genome, and anti-MPXV drug screening (Figure 1). In short, the first part is the analysis of mutations and PSSs within the MPXV genome, which aims to obtain high-quality MPXV sequences and uncover mutations and PSSs within the MPXV genome. The second part is the screening of anti-MPXV drugs. MIHPs were identified by the collected PPIs, and some analysis was performed on them. Finally, the drugs that targeted MIHPs and MPXV proteins were screened and were further evaluated.

### 2.2. Data Preparation

Complete high-quality MPXV genomic sequences were downloaded from the GISAID database [28]. After the quality control, Python script was written to remove the sequence containing 15 consecutive bases (N) that could not be detected. The sequences were classified according to the lineages.

### 2.3. Identification of Homologous Genes

Identification of homologous genes was performed by tblastn in BLAST (Version 2.15.0) [29]. Due to the high conservation of the MPXV protein, the e-values less than 1 × e^−10^ were set to ensure the accuracy of the results. According to the identification results of tblastn, a script written by Python was used to extract and classify the proteins from the original sequence.

### 2.4. Analysis of Mutation

After the removal of the termination codon, the MPXV protein sequences were subjected to multiple sequence alignment using MAFFT (Version 7.149) [30]. Mutation analysis was performed using the mutation analysis module in BioAider (Version 1.527) [31].

### 2.5. Analysis of Selective Pressure

The methods used to investigate PSSs included Single Likelihood Ancestor Counting (SLAC), Fixed Effects Likelihood (FEL), Mixed Effects Model of Evolution (MEME), and Fast Unconstrained Bayesian AppRoximation (FUBAR). FUBAR in the Datamonkey website (https://www.datamonkey.org/, accessed on 2 September 2023) was used to detect PSSs [32]. When the posterior probability was greater than 0.9, we proposed the site was under selective selection.

### 2.6. Visualization of Non-Synonymous Mutations and PSSs

The PDB database (https://www.rcsb.org/) and AlphaFold2 online site (https://alphafold.ebi.ac.uk/, accessed on 2 October 2023) were used to obtain the 3D structures of protein, and ChimeraX (Version 1.5) was used to visualize the non-synonymous mutations and PSSs [33,34,35].

### 2.7. Construction and Visualization of the PPI Network

The PPI data between viruses and human were collected from the STING database (https://www.string-db.org/, accessed on 15 October 2023), Virus.STING database (http://viruses.string-db.org, accessed on 27 October 2023), PubMed database (https://pubmed.ncbi.nlm.nih.gov/, accessed on 23 October 2023), and HPIDB database (https://cales.arizona.edu/hpidb/about.html, accessed on 5 November 2023) [36,37,38]. A total of 415 PPIs were collected from the four databases. (1) A total of 32 PPIs were collected in the Virus.STRING database according to the homologous proteins of MPXV in other poxviruses; (2) 330 PPIs were manually selected from 2137 articles downloaded from PubMed database; (3) the 179 protein sequences of MPXV were entered into the HPIDB database to predict 369 PPIs, and 49 PPIs were obtained after removing duplicate PPIs; (4) all viral proteins that can interact with human proteins were downloaded from the Virus.STING database and treated as a protein library. MPXV proteins with e-values less than 0.001 and sequence consistency and coverage greater than 0.3 were selected by using blastp, and these MPXV proteins were regarded as homologous proteins of other viral proteins that can interact with human protein in the protein library above, with a total of 4 PPIs collected. The PPI network was visualized under the help of the “yFiles Organic Layout” style in the “Layout” module of Cytoscape (version 3.7.1) [39].

### 2.8. Functional Enrichment Analysis

The Gene Ontology (GO) terms, Kyoto Encyclopedia of Genes and Genomes (KEGG) pathways, the Reactome pathway knowledgebase, and WikiPathways pathways were conducted with functions of enrichGO(), enrichKEGG(), enrichier(), and enrichPathway() in the R package clusterProfiler (version 4.0.3) [40]. All functional enrichment analyses with adjusted *p*-values less than 0.01 were considered significant.

### 2.9. Prediction of Antiviral Drugs

Candidate drugs were predicted under the help of the DrugBank database (https://www.drugbank.com/, accessed on 11 December 2023) [41]. The protein sequences of MIHPs and MPXV proteins were queried against DrugBank for similar targets with the default parameters.

### 2.10. Molecular Docking and Visualization

The PDB database (https://www.rcsb.org/, accessed on 16 January 2024) and AlphaFold protein structure database (https://alphafold.ebi.ac.uk/, accessed on 16 February 2024) were used to obtain PDB-formatted protein structure files [33,34]. SDF-formatted drug structure files were obtained from the PubChem database (https://pubchem.ncbi.nlm.nih.gov/, accessed on 17 February 2024) and converted into PDB-formatted protein structure files through OpenBabel (version 3.1.1) [42,43]. AutoDock (version 4.0) was used to perform molecular docking, the optimal docking sites were selected, and the docking results were visualized by ChimeraX (Version 1.5) [35,44].

## 3. Results

### 3.1. Detection Mutations in MPXV Genome

**Figure 2:**
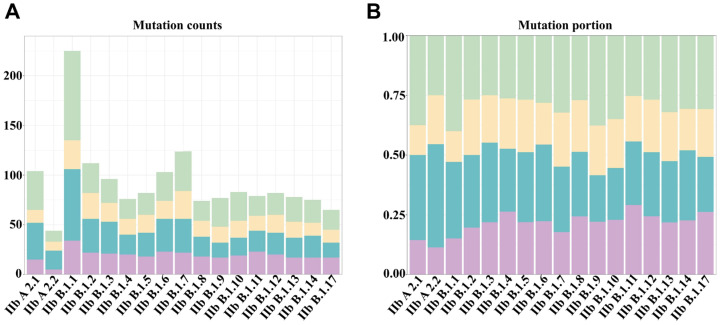
The nonsynonymous mutations and synonymous mutations in different lineages of MPXV. (**A**) The number of nonsynonymous mutations and synonymous mutations in the core and variable regions of the MPXV genome. (**B**) The proportion of nonsynonymous mutations and synonymous mutations in the core and variable regions of the MPXV genome. Green indicates nonsynonymous mutations in the core regions; yellow indicates synonymous mutations in the core regions; blue indicates non-synonymous mutations in the variable regions; purple indicates synonymous mutations in the variable regions.

A total of 5089 MPXV sequences were downloaded, and after quality control, 1068 genomic sequences were used for further analysis (Supplementary Table S1). The average genome size of MPXV was 197,485 bp, with an average GC content of 32.9% (Supplementary Figure S1). We conducted a mutation analysis of MPXV proteins, identifying a total of 538 non-synonymous mutations, 253 synonymous mutations, and 8 base deletions, with no base insertions compared to the first clade in the 2022 outbreak (Supplementary Table S2). Additionally, six non-synonymous mutations and three synonymous mutations occurred in the F13L (NBT03_gp037) gene, while six non-synonymous mutations and six synonymous mutations were found in the E9L (NBT03_gp050) gene (Supplementary Figure S2). To explore mutations across different lineages, we counted the synonymous and non-synonymous mutations in the core and variable regions of all MPXV lineages. We detected that the mutation rate in the variable regions was higher than in the core regions. Notably, Clade IIb B.1.1 exhibited the largest number of mutations, with a higher proportion of non-synonymous mutations in the core regions compared to the variable regions. In contrast, Clade IIb A.2.2 had the fewest mutations, showing a greater proportion of non-synonymous mutations in the variable regions compared to the core regions (Figure 2).

### 3.2. PSSs in MPXV Genes

**Figure 3:**
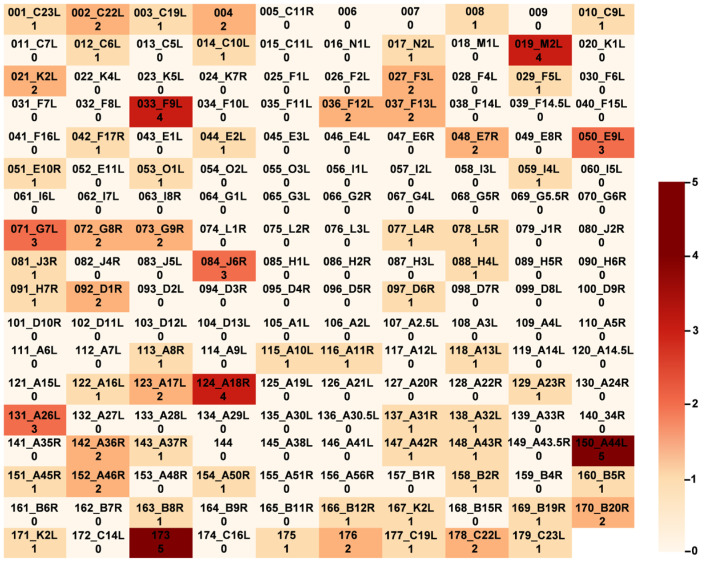
PSSs of MPXV genes. For each square, the first row is the name of the genes, and the second row is the number of PSSs. The darker the color, the more PSSs. A total of 40 PSSs in MPXV genes were detected. The genes encoding these proteins are C19L, NBT03_gp004, NBT03_gp006, NBT03_gp008, C5L, N2L, M2L, F3L, F5L, F6L, F12L, E2L, E7R, G6R, G7L, G9R, D1R, D8L, A3L, A4L, A17L, A26L, A35R, A43R, A44L, B4R, B6R, B20R, NBT03_gp173, and NBT03_gp176. Among them, G7L exhibited the highest number of PSSs. PSSs, positive selection sites.

To further screen the sites under positive selection during evolution, we conducted a positive selection analysis of MPXV genes. A total of 40 PSSs in MPXV genes were detected. The genes encoding these proteins are C19L, NBT03_gp004, NBT03_gp006, NBT03_gp008, C5L, N2L, M2L, F3L, F5L, F6L, F12L, E2L, E7R, G6R, G7L, G9R, D1R, D8L, A3L, A4L, A17L, A26L, A35R, A43R, A44L, B4R, B6R, B20R, NBT03_gp173, and NBT03_gp176. Among them, G7L exhibited the highest number of PSSs (Figure 3). Furthermore, compared to the first clade in the 2022 outbreak, 20 (50%) non-synonymous mutations were observed in the PSSs, including E28V in C5L, N43L in E7R, and N44F in E7R.

### 3.3. The Impact of Mutations and PSSs on Protein Structure

**Figure 4:**
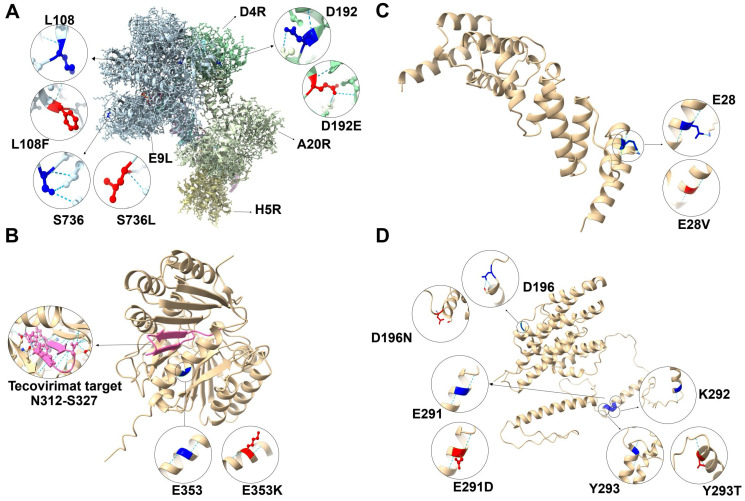
Visualization of nonsynonymous mutations and PSSs in the MPXV protein. (**A**) Visualization of nonsynonymous mutations in the E9L-D4R-A20R-H5R complex. The light blue region is the 3D structure of E9L; the dark green region is the 3D structure of D4R; the light green region is the 3D structure of A20R; the light-yellow region is the 3D structure of H5R. (**B**) Visualization of nonsynonymous mutations in the F13L. The pink region is the targeted region of tecovirimat. (**C**) Visualization of PSGs and nonsynonymous mutations in C5L. (**D**) Visualization of PSSs and nonsynonymous mutations in G7L. Blue is the original structure; red is the structure after mutation. The dashed line in light blue shows hydrogen bonds; atoms and bonds within the 5.0 Å range of distance mutations in the ball-and-stick model are colored by element, with oxygen atoms shown in red and nitrogen atoms shown in blue.

Among the 538 non-synonymous mutations and 40 PSSs identified, we selected the E9L-D4R-A20R-H5R complex, F13L, C5L, and G7L for mutational visualization analysis based on the significance of known functional genes of MPXV (Figure 4). The 3D structure of the E9L-D4R-A20R-H5R complex has been resolved (PDB serial number: 8WPE). In contrast, the 3D structures of F13L, C5L, and G7L have not been resolved, so we predicted their structures and selected the models with the highest scores for display (Supplementary Table S3).

Replication of MPXV relies on the E9L-D4R-A20R-H5R complex. Within this complex, the L108F mutation in E9L was observed in Clades IIb B.1.1 to B.1.17, the S736L mutation appeared in Clade IIb B.1.1 l, the D192E mutation in D4R was found in Clade IIb A.2.1, and the S92F mutation in H5R was noted in Clade IIb B.1.10. Due to the unresolved structure of S92, it could not be visualized. In the complex, L108 is located on the surface of E9L, D192 is on the surface of D4R, and S736 is within the chain of E9L. The hydrogen bond structure did not change after the L108F mutation; however, a new hydrogen bond to E188 was introduced following the D192E mutation. After the S736L mutation, E9L lost the two hydrogen bonds associated with E773 and R776 (Figure 4A).

F13L is the target protein of tecovirimat. The E353K mutation in F13L was observed in Clade IIb, specifically from B.1.1 to B.1.17. E353 is located on the surface of F13L, and the E353K mutation did not alter the hydrogen bond structure (Figure 4B).

Among all viral genomes, only the poxvirus genome has been reported to encode the KBTBD protein C5L. The E28V mutation on the surface of C5L was found in Clade IIb B.1.1. Following the E28V mutation, the hydrogen bond connecting to K17 was lost, and the structure associated with the nitrogen atom of K17 was disrupted (Figure 4C).

There were three consecutive PSSs in G7L (E291, K292, and Y293). The mutations E291D, K292A, and Y293T appeared in only two sequences of Clade IIb B.1.1, while E291G, K292A, and Y293T were found in only one sequence of Clade IIb B.1.1. Additionally, G7L had a non-synonymous mutation site at D196N, which was identified in Clade IIb B.1.1 to B.1.17. However, the E291G and K292A mutations could not be visualized. The D196 residue was located within the G7L chain, and following the D196N mutation, two hydrogen bonds connecting to S198 were lost, disrupting the connection to the oxygen atom of S198. E291, K292, and Y293 were all located on the surface of G7L, and the E291D and Y293T mutations did not alter the hydrogen bond (Figure 4D).

### 3.4. PPIs Between MPXV and Human

After manual data screening and homologous comparison, 415 PPIs were collected from four databases (Supplementary Table S4). The network comprised 64 MPXV proteins and 346 MPXV-interacting human proteins (MIHPs). Notably, among the MPXV proteins, E3L (OPG065), C6L (OPG029), K7R (OPG044), and K2L (OPG199) each interacted with more than 40 MIHPs (Supplementary Figure S3).

### 3.5. Interaction Network Between MIHPs and Other Viruses

**Figure 5:**
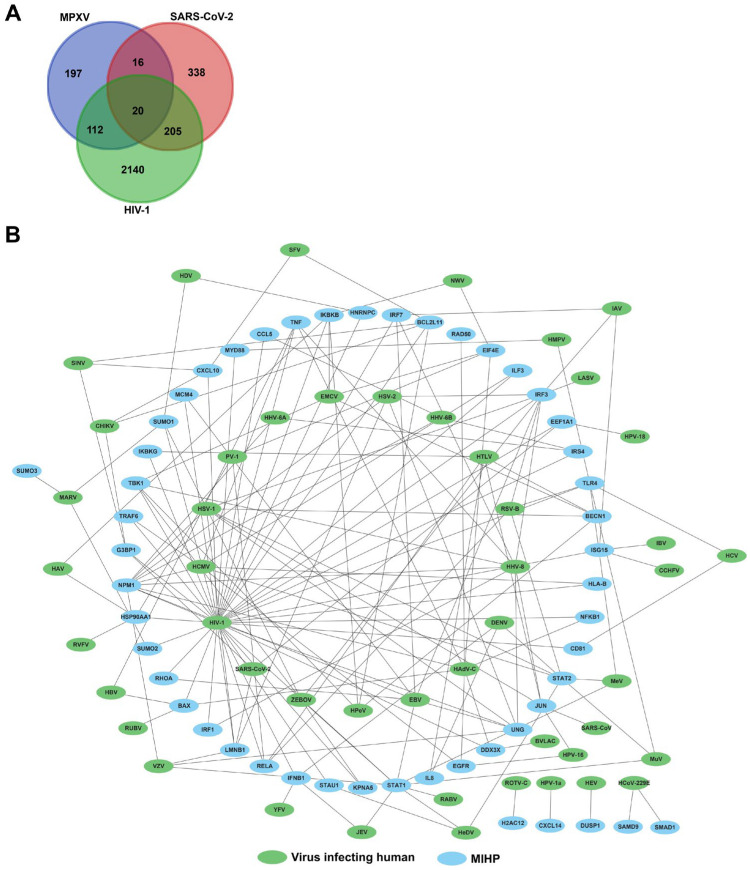
MIHPs interact with other viruses infecting humans. (**A**) Venn diagram of human proteins interacting with MPXV, SARS-CoV-2, and HIV-1. A total of 36 MIHPs interacting with SARS-CoV-2, 132 MIHPs interacting with HIV-1, and 20 MIHPs interacting with both SARS-CoV-2 and HIV. (**B**) The interaction network between MIHPs and other viruses infecting humans. A total of 159 MIHPs interacted with other viruses, and 60 MIHPs interacted with more than one virus. MIHP, MPXV-interacting human proteins.

To explore the interactions between MIHPs and other viruses that infect humans, we constructed an interaction network of them (Figure 5). Considering that patients infected with MPXV can also be co-infected with both SARS-CoV-2 and HIV, we collected and screened the PPIs between viral proteins (SARS-CoV-2 and HIV) and MIHPs. We identified 36 MIHPs interacting with SARS-CoV-2, 132 MIHPs interacting with HIV-1, and 20 MIHPs interacting with both SARS-CoV-2 and HIV (Figure 5A). Additionally, 159 (46.0%) MIHPs interacted with other viruses, and 60 (17.3%) MIHPs interacted with more than one virus; for example, NPM1 interacted with ten other viruses simultaneously (Figure 5B, Supplementary Table S5).

### 3.6. Functional Enrichment Analysis of MIHPs

To explore the functions of the 346 MIHPs, we conducted a functional enrichment analysis and identified the top ten items (Supplementary Figure S4). In the GO terms, the analysis of biological processes revealed that eight processes were associated with the host’s innate immune response, while the remaining two were linked to the assembly of protein complexes. In terms of cellular components, three terms were related to the cytoskeleton, three were related to the vesicular region within the cell, and two were related to chromosomal structure and the process of mitosis. The final two terms identified were “Cytoplasmic stress granule” and “Cornified envelope” (Supplementary Figure S4A).

In the KEGG pathway enrichment analysis, six pathways were associated with disease, including Salmonella infection. Two pathways were related to host autoimmunity, including neutrophil extracellular trap formation and NOD-like receptor signaling pathway. The remaining two pathways were enriched for alcoholism and necroptosis (Supplementary Figure S4B).

The Reactome enrichment analysis identified five signaling pathways related to cell proliferation, migration, and division, including RHO GTPase effectors. Additionally, five pathways were associated with viral infections and host innate immune responses, particularly those related to cytomegalovirus and acute respiratory syndrome (Supplementary Figure S4C).

In the WikiPathways enrichment analysis, five signaling pathways involved in cell proliferation, differentiation, and apoptosis were identified, such as the chemokine signaling pathway. The remaining five pathways were related to innate immune regulation of the host, including the Toll-like receptor signaling pathway (Supplementary Figure S4D).

### 3.7. Predicting Anti-MPXV Drugs Targeting MIHPs and MPXV Proteins

A total of 403 drugs targeting 140 (40.5%) MIHPs were identified, of which 174 (43.1%) have been approved for the treatment of other diseases. Among the 403 drugs, 370 (91.8%) can directly interact with MIHPs, while the remaining drugs interact with analogues of MIHPs (Supplementary Table S6). Drugs with no medicinal value were excluded from consideration, including those in the research stage, withdrawn drugs, and those that provide only nutritional value (Supplementary Figure S5A). Since alvespimycin (DB12442) and imiquimod (DB00724) have the potential to treat poxvirus, both were screened as candidate anti-MPXV drugs targeting MIHPs.

A total of 117 drugs targeting MPXV proteins were identified: 64 (54.7%) had been approved for the treatment of other diseases, and 28 (23.9%) interacted with both MIHPs and MPXV proteins. Of these, only ten (8.54%) predicted drugs can interact directly with MPXV proteins, including E9L, F13L, and J3R. The remaining drugs interact with analogues of MPXV proteins (Supplementary Figure S5B and Table S7). Among E9L, F13L, and J3R, only J3R exhibits immunogenicity. Consequently, eight compounds that directly target J3R were screened as potential anti-MPXV drugs. These include S-adenosyl-L-homocysteine (DB01752), 7-methyl-7,8-dihydroguanosine-5’-diphosphate (DB01960), 7,9-dimethylguanine (DB01978), 6-amino-1-methyl-7H-purin-1-ium (DB03164), 7-methyl-7,8-dihydroguanosine-5’-(tetrahydrogen triphosphate) (DB03358), 7-methylguanosine (DB03493), 3-methylcytosine (DB04103), and 1-methylcytosine (DB04314).

Since the eight compounds targeting J3R were still in the experimental stage, their physicochemical properties were used to roughly evaluate their druglikeness. The evaluation indicators we adopted were the Rule of 5 (Ro5), water solubility (WS), and molecular solubility (LogS). We found that the RBs of these eight compounds were less than ten, and LogPs were less than five, which conformed to both rules of Ro5. However, the molecular weight of 5’-(tetrahydrogen triphosphate) exceeded 500 g/mol, and its hydrogen bond donor (HBD) and hydrogen bond acceptor (HBA) both surpassed the Ro5 thresholds. Additionally, the HBA and HBD values of S-adenosyl-L-homocysteine and 7-methylguanosine only met the Ro5 thresholds, resulting in poorer druglikeness for these three compounds (Table 1). Currently, it is considered that good docking is achieved when the binding energy is less than −1.2 kcal/mol. Therefore, 7-methyl-7,8-dihydroguanosine-5’-diphosphate and 5’-(tetrahydrogen triphosphate) exhibited poor binding with J3R (Supplementary Table S8).

**Figure 6:**
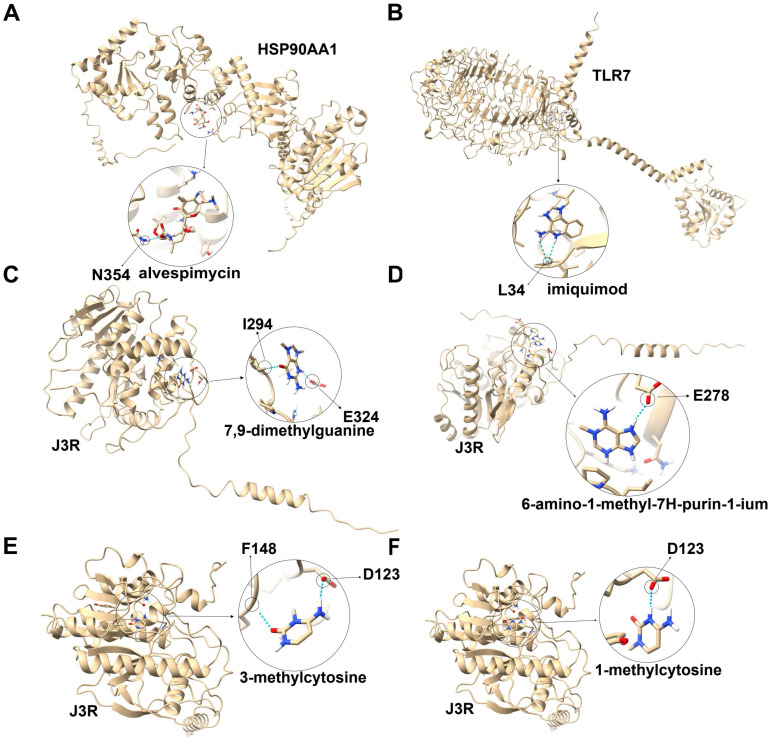
Interaction model between candidate anti-MPXV drugs and their targeted proteins. (**A**) Interaction model between HSP90AA1 and alvespimycin. (**B**) Interaction model between TILR7 and imiquimod. (**C**) Interaction model between J3R and 7,9-dimethylguanine. (**D**) Interaction model between J3R and 6-amino-1-methyl-7h-purine-1-ium. (**E**) Interaction model between J3R and 3-methylcytosine. (**F**) Interaction model between J3R and 1-methylcytosine. In the ball-and-stick model, the dashed line in light blue represents hydrogen bond; red atom indicates oxygen; blue atom indicates nitrogen; white atom indicates hydrogen; yellow atom indicates sulfur; orange atom indicates phosphorus.

After screening, alvespimycin and imiquimod were predicted to be anti-MPXV drugs targeting MIHPs. Additionally, 7,9-dimethylguanine, 6-amino-1-methyl-7H-purin-1-ium, 3-methylcytosine, and 1-methylcytosine were predicted as anti-MPXV drugs targeting MPXV proteins. Alvespimycin formed one hydrogen bond with the N354 of HSP90AA1 (Figure 6A), while imiquimod formed one hydrogen bond with the L34 of TLR7 (Figure 6B). 7,9-Dimethylguanine established one hydrogen bond with the I294 and E324 of J3R (Figure 6C). 6-Amino-1-methyl-7H-purin-1-ium formed one hydrogen bond with E278 of J3R (Figure 6D). 3-Methylcytosine bonded to J3R through a hydrogen bond at D123 and F148 (Figure 6E). Finally, 1-methylcytosine was bonded to J3R through a hydrogen bond at D123 (Figure 6F).

## 4. Discussion

Mutations are the building blocks of most evolution [45]. Understanding the mutational patterns of MPXV will help us monitor its epidemiology and evolution. We detected 799 mutations occurred in the MPXV, with mutation rates in the variable regions being higher than those in the core regions, which aligns with previously reported results [18]. The proportions of base deletions and insertions were small, likely because they can disrupt the proper expression of viral genes. For example, the K3L host range gene of MPXV has numerous base deletions that hinder accurate expression [46]. Additionally, mutations in the tecovirimat-targeting protein F13L and the brincidofovir-targeting protein E9L may contribute to drug resistance [47]. MPXV replication is primarily mediated by E9L DNA polymerase, which requires the A20R processivity factor, D4R uracil-DNA glycosidase, and H5R phosphorylated protein [48]. The D192E mutation of D4R and the S736L mutation of E9L could alter the replication capacity of MPXV. We identified 40 PSSs in MPXV genes. Notably, PSSs were found in C5L, which has been shown to be uniquely encoded by the poxvirus genome [49]. These mutations and PSSs lead to changes in the hydrogen bond structure, which play an integral role in maintaining protein stability [50]. Therefore, these mutations and PSSs may result in structural changes in proteins, potentially affecting the virulence and transmission of MPXV.

Currently, there is no specific treatment for MPXV infection [51]. Our understanding of the pathogenesis of MPXV is insufficient, limiting the development of anti-MPXV drugs [52]. Drug prediction is crucial for drug development, making it particularly important to identify potential anti-MPXV drugs. Through the PPIs network, we identified 346 MIHPs. We conducted functional enrichment analysis to identify the biological processes associated with MIHPs, allowing us to select drugs that target these processes to treat MPXV infection. For example, recently, three peptides were designed to inhibit the migration and adhesion of MPXV in vivo by blocking the binding of A36R of MPXV to human actin [53]. Among the predicted drugs targeting MIHPs, alvespimycin and imiquimod show potential for treating poxvirus [54,55,56,57]. Among the predicted drugs targeting MPXV proteins, only J3R exhibits immunogenicity [58]. Therefore, alvespimycin and imiquimod were predicted as anti-MPXV drugs targeting MIHPs. Additionally, 7,9-dimethylguanine, 6-amino-1-methyl-7H-purin-1-ium, 3-methylcytosine, and 1-methylcytosine were predicted as anti-MPXV drugs targeting the MPXV protein J3R. MIHPs may interact with other viruses that infect humans, suggesting that drugs targeting MIHPs could have a broad spectrum of activity. Alvespimycin is a new generation of heat shock protein 90 (Hsp90) inhibitor and has also been found to regulate cell cycle genes [59,60]. This regulation may inhibit the replication of MPXV and other viruses by disrupting the cell cycle. Imiquimod induces the synthesis of IFN-α and pro-inflammatory Th1 cytokines [61], leading us to speculate that imiquimod may treat MPXV and other viral infections by modulating the innate immune response. Since viral proteins mutate at a much faster rate than host proteins, compared with cidofovir-, brincidofovir-, and tecovirimat-targeted poxvirus proteins, alvespimycin and imiquimod targeting MIHPs may be more stable and less susceptible to drug resistance.

## 5. Conclusions

In summary, we uncovered a total of 799 mutations and 40 PSSs within the MPXV genome. We found that these mutations and PSSs potentially impact protein structure. We identified 346 MIHPs, which interacted with other viruses and were significantly enriched in host immunity pathways. Finally, we identified two drugs and four compounds as promising candidates against MPXV. This pure information analysis research has limitations. Firstly, further experiments are needed to verify whether the mutations and PSSs mentioned above can change the replication or immune escape capabilities of MPXV. Secondly, the constructed PPIs between MPXV and human proteins are not comprehensive enough, which limits the predicted drugs. Additionally, the compounds we identified that target MPXV proteins only exhibit druglikeness properties, and their developability requires further experimental assessment in future research.

## Figures and Tables

**Table 1:** Physicochemical properties of the eight drugs interacting with J3R.

Drug	MW (g/mol)	RB	HBD	HBA	LogP	WS (mg/ML)	LogS
DB01752	384.41	7	5	10	−3.5	4.08	−2
DB01960	459.24	6	7	14	−5	7.91	−1.8
DB01978	179.18	0	1	4	0.5	3.14	−1.8
DB03164	150.16	0	2	3	−0.1	4.33	−1.6
DB03358	539.22	8	8	17	−6.1	/	/
DB03493	298.28	2	5	6	−1.8	5.61	−1.8
DB04103	125.13	0	1	2	−1	11.5	−1.2
DB04314	125.13	0	1	1	−1.1	29.1	−0.63

“/” indicates no physicochemical properties of the drug. MW, molecular weight; RB, rotatable bond; HBD, H-bond donor; HBA, H-bond acceptor; LogP, lipid–water partition coefficient; WS, water solubility; LogS, molecular solubility. Interaction model between candidate anti-MPXV drugs and their targeted proteins.

## Data Availability

The original contributions presented in the study are included in the article and Supplementary Materials, further inquiries can be directed to the corresponding author.

## Supplementary Materials

The following supporting information can be downloaded at https://www.mdpi.com/article/10.3390/microorganisms12112239/s1. Figure S1: The size and GC content of the MPXV genome. Figure S2: Heatmap of mutations of MPXV proteins. Figure S3: PPIs between MPXV and human. Figure S4: Functional enrichment analysis of MIHPs. Figure S5: Prediction of anti-MPXV drugs. Table S1: MPXV sequences downloaded from the GISAID database. Table S2: The mutation analysis of the MPXV. Table S3: Scores of each protein prediction model. Table S4: PPIs between MPXV and human and their source. Table S5: Other viruses infecting humans that interact with MIHPs. Table S6: Drugs targeting MIHPs. Table S7: Drugs targeting MPXV proteins. Table S8: Binding energy of candidate anti-MPXV drugs and their targeted proteins.
