# Supplementary material for: Evolutionary Analysis and Antiviral Drug Prediction of Mpox Virus
Source: Microorganisms. 2024 Nov 5;12(11):2239. doi: 10.3390/microorganisms12112239 (PMC11596041; doi:10.3390/microorganisms12112239)
Supplement: Supplementary file 1 [file microorganisms-12-02239-s001.zip › Supplementary Figures.pdf]

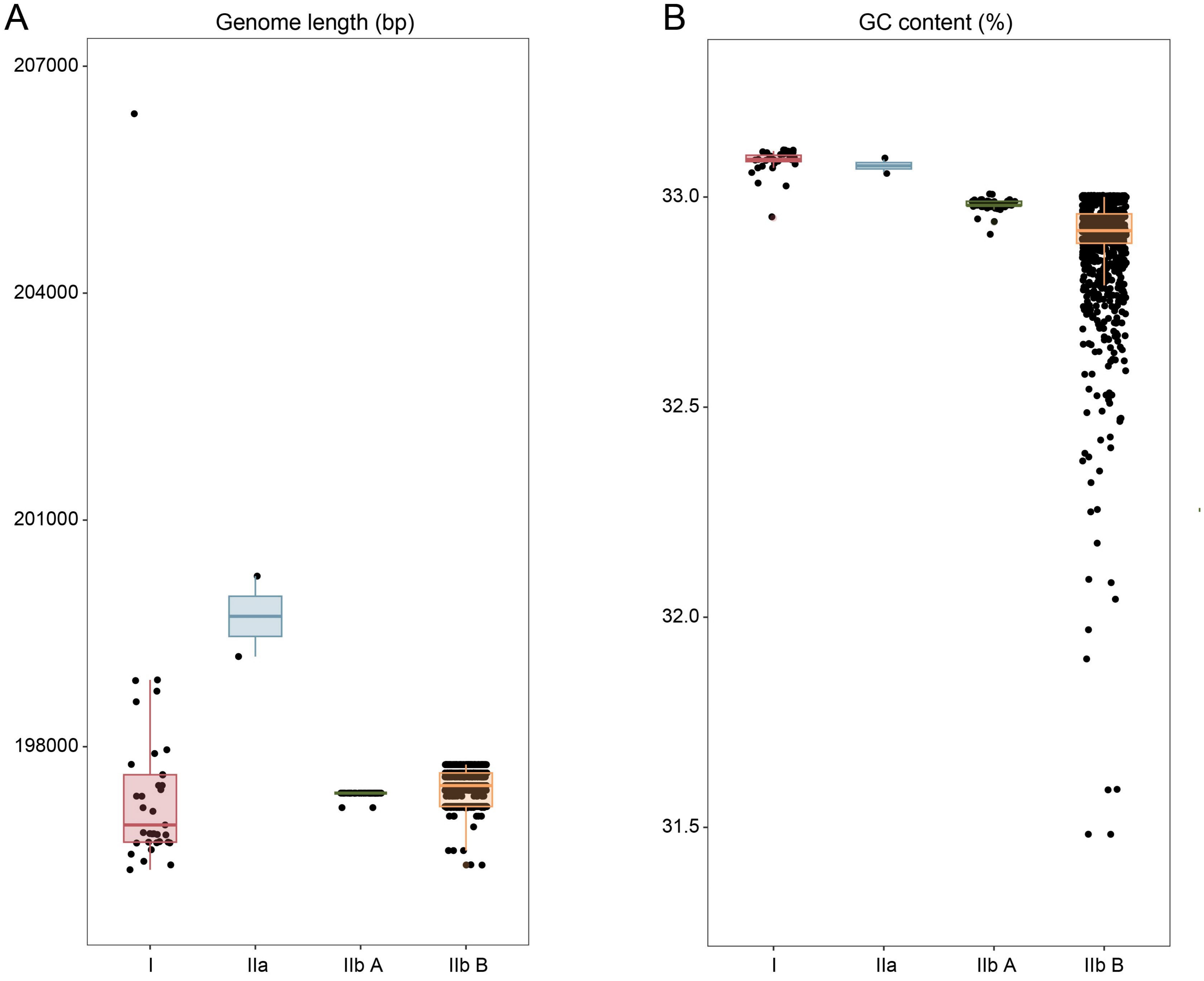

Supplementary Figure 1. The size and GC content of MPXV genome. (A)The size of MPXV genome. (B) The GC content of MPXV genome.

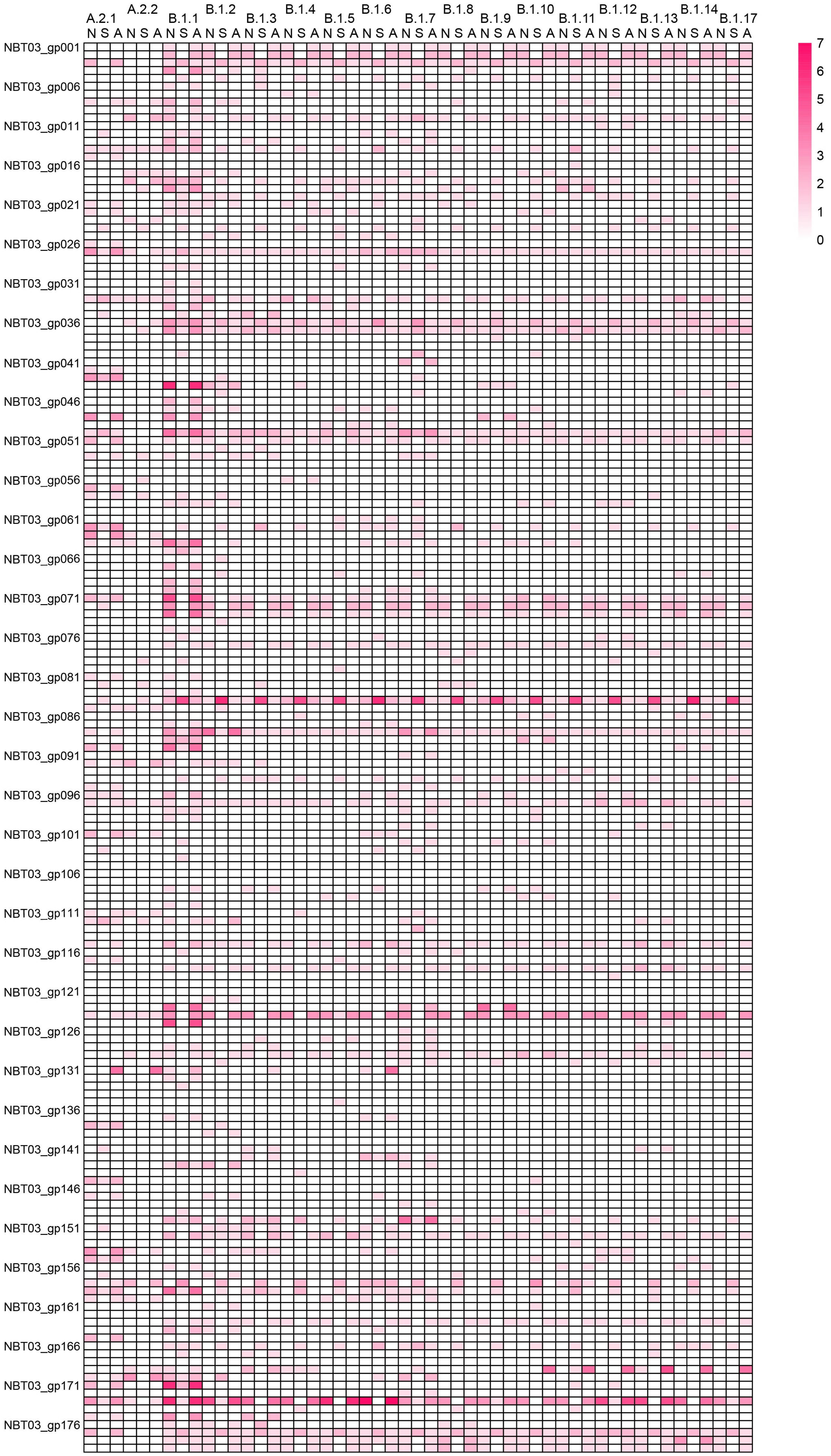

Supplementary Figure 2. Heatmap of mutations of MPXV proteins. N indicates nonsynonymous mutation; S indicates synonymous mutation; A indicates amino acid mutation.

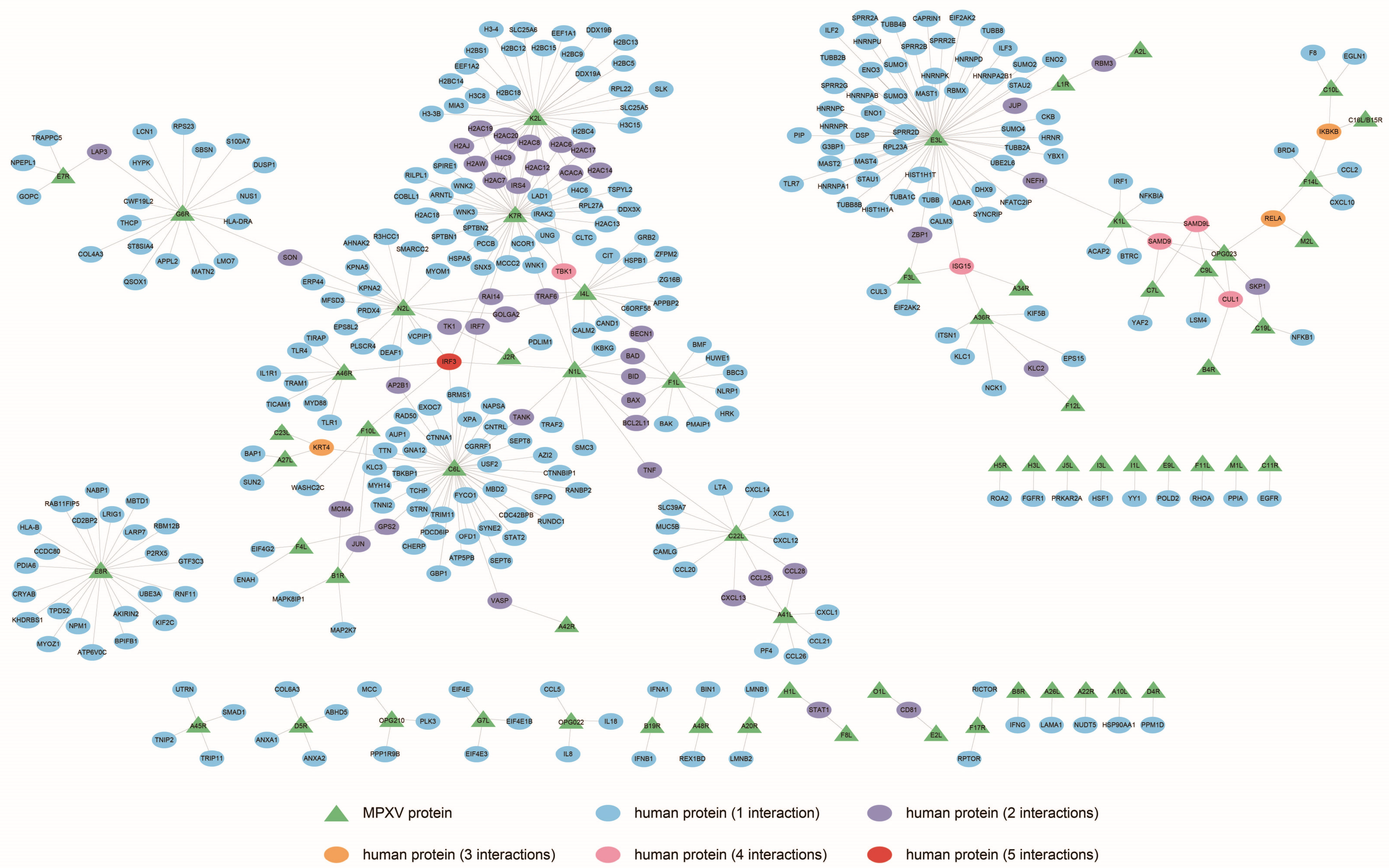

Supplementary Figure 3. PPIs between MPXV and human. The green triangle represents MPXV protein; the blue oval represents human protein that interacts with one MPXV protein; the purple oval represents human protein that interacts with two MPXV proteins; the orange oval represents human protein that interacts with three MPXV proteins; the pink oval represents human protein that interacts with four MPXV proteins; the red oval represents human protein that interacts with five MPXV proteins.

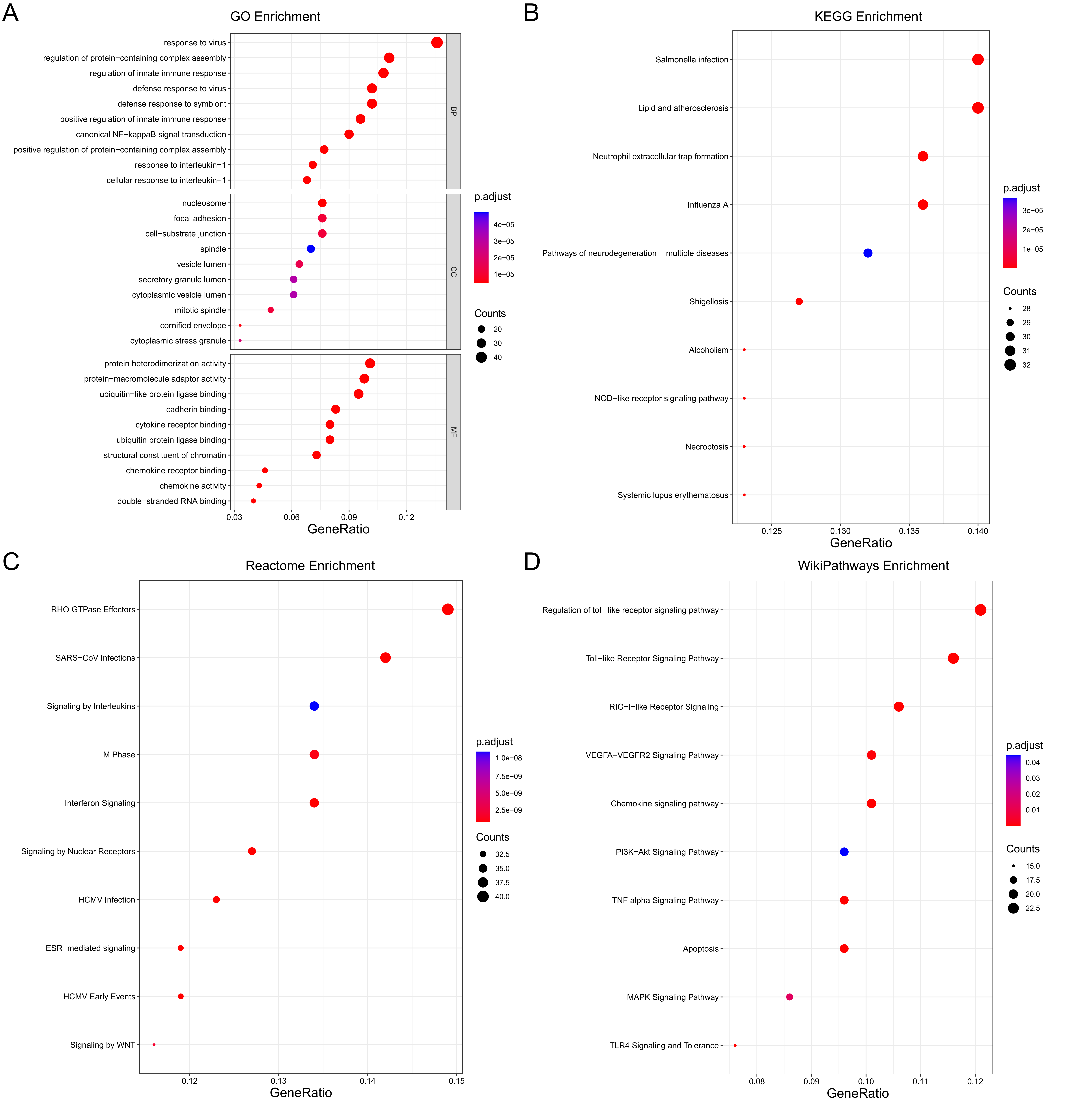

Supplementary Figure 4. Functional enrichment analysis of MIHPs. (A) GO enrichment analysis. (B) KEGG enrichment analysis. (C) Reactome enrichment analysis. (D) WikiPathways enrichment analysis. Each functional enrichment analysis lists only the top ten enriched terms.

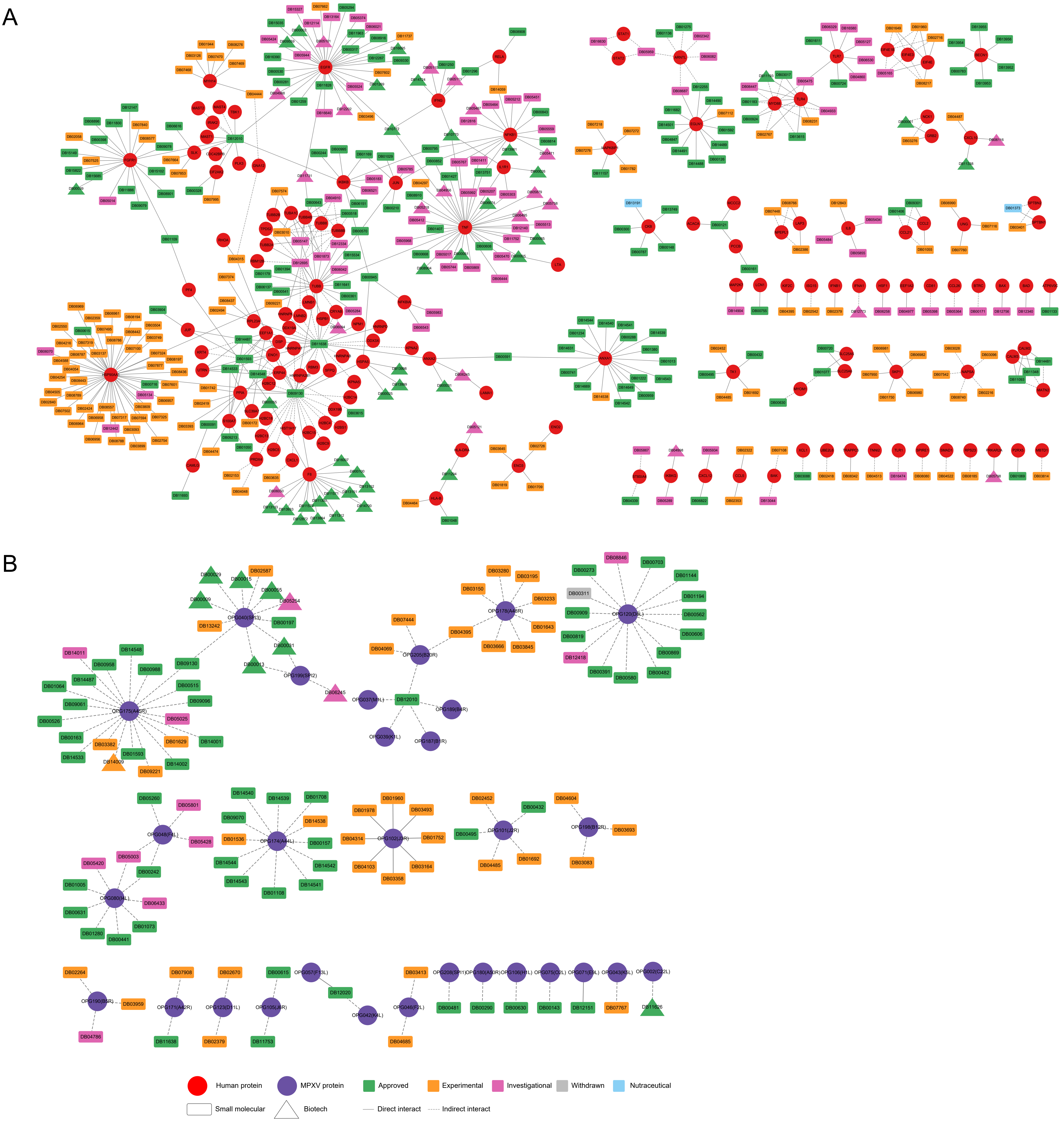

Supplementary Figure 5. Prediction of anti-MPXV drugs. (A) Interactions network between MIHPs and antiviral drugs. (B) Interactions between MPXV and antiviral drugs. Red circle represents human protein; purple circle represents the MPXV protein; rectangle represents small molecule drug; the triangle represents biotech drug. Green indicates drug that has been approved for use; orange indicates drug that is still in the experimental stage; pink indicates drug that is still in the research stage; blue indicates drug that has only nutritional effects; gray indicates drug that has been withdrawn. The solid line indicates that the drug can directly interacts with the protein; the dashed line indicates that the drug cannot interacts directly with proteins but interacts with their analogues.
